# Salvage therapy in cancer patients with hepatitis C without sustained virologic response after direct‐acting antivirals—A prospective study

**DOI:** 10.1002/jgh3.12294

**Published:** 2019-12-19

**Authors:** Haley Pritchard, Deeksha Jandhyala, Jeff Hosry, Georgios Angelidakis, Harrys A Torres

**Affiliations:** ^1^ Department of Infectious Disease Baylor College of Medicine Houston Texas USA; ^2^ Department of Infectious Diseases, Infection Control and Employee Health The University of Texas MD Anderson Cancer Center Houston Texas USA; ^3^ Department of Gastroenterology, Hepatology, and Nutrition The University of Texas MD Anderson Cancer Center Houston Texas USA

**Keywords:** cancer, direct‐acting antivirals, hepatitis C virus, salvage therapy

## Abstract

**Background and Aim:**

No information exists regarding direct‐acting antivirals (DAAs) salvage therapy for Hepatitis C (HCV)‐infected patients with any type of cancer. We prospectively evaluated the safety and efficacy (SVR12) of salvage therapy in these patients.

**Methods:**

Patients who failed initial DAAs (01/2015–01/2018) were analyzed. Resistance‐associated substitutions to NS5A and NS3 were investigated by population sequencing.

**Results:**

Of 164 patients enrolled, 16 (10%) experienced treatment failure. Of these, 11 patients received salvage therapy. The majority (91%) were men; 55% had genotype 1a, 45% had cirrhosis, and 45% had hepatocellular carcinoma. Four patients failed the first salvage therapy, and two of them required a second salvage therapy. Overall, 9 of 11 (82%) patients achieved SVR12. All four patients treated with sofosbuvir/velpatasvir/voxilaprevir (+/− ribavirin) achieved SVR12. The presence of resistance‐associated substitutions did not impact response. Seven patients developed grade 1/2 adverse events. No patient had grade 3/4 adverse events. No patient required interruption of DAA therapy because of clinical or laboratory abnormalities.

**Conclusions:**

This is the first prospective study in HCV‐infected cancer patients failing DAAs. The efficacy of salvage therapy in this group appears to be lower than previously reported in non‐cancer patients, but better response rates are observed with newer regimens. Salvage therapy is associated with minimal toxicity.

## Graphical abstract

This is the first prospective study in HCV‐infected cancer patients failing direct‐acting antivirals. The efficacy of salvage therapy in this group appears to be lower than the previously reported in non‐cancer patients but better response rates are observed with newer regimens. Salvage therapy is associated with minimal toxicity.

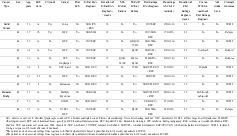

## Introduction

The rate of sustained virologic response (SVR) to a regimen of at least two direct‐acting antiviral (DAA) approaches 95–99% in treatment‐naïve patients with Hepatitis C virus (HCV) infection across genotypes 1–6.^1^ Retreatment can be challenging as exposure to DAAs can induce resistance‐associated substitutions (RASs) that can persist for many months after initial exposure.^2^ The development of RASs decreases the rate of SVR after subsequent DAA treatment and necessitates a longer duration with increased pill burden and the potential use of ribavirin associated with significant toxicity.

Elimination of HCV from infected cancer patients leads to better hepatic, virologic, and oncologic outcomes.^3^, ^4^ HCV eradication improves transaminase levels and delays the progression of liver fibrosis, enabling patients to receive hepatotoxic chemotherapies.^3^ Virologic clearance of HCV also improves overall survival, improves disease‐free survival, reduces relapses of HCV‐associated non‐Hodgkin lymphoma, and prevents secondary HCV‐associated cancer.^3^ HCV infection is an exclusion criterion in many clinical trials, and virologic cure of HCV may give patients more opportunities for clinical trials of treatment for their primary malignancy.^3^ However, there are no published data regarding the retreatment of HCV‐infected cancer patients with DAAs. Here, we report our experience with salvage therapy in HCV‐infected cancer patients without SVR to DAA therapy.

## Methods

As a part of an ongoing prospective observational study, we enrolled 164 HCV‐infected cancer patients undergoing DAA treatment at the University of Texas M.D. Anderson Cancer Center between January 2015 and January 2018. The study was approved by the Institutional Review Board of MD Anderson Cancer Center, and patients provided written informed consent. DAA therapy was offered to all cancer patients without contraindication for antiviral therapy as previously reported.^3^ Treatment was not offered to patients with a life expectancy of <12 months that cannot be remediated by HCV treatment or cancer therapy or to patients with uncontrolled cancer unless the patient was a candidate for a cancer clinical trial.^3^ Patients without SVR after initial treatment were offered a first DAA salvage regimen with or without ribavirin, and if that regimen failed, a second regimen as salvage therapy was used per the American Association for the Study of Liver Diseases/Infectious Diseases Society of America (AASLD/IDSA) HCV treatment guidelines.^1^ Baseline assessments included standard clinical laboratory testing, measurement of serum HCV RNA levels, and determination of HCV genotype. HCV RNA levels were measured using COBAS AmpliPrep or COBAS TaqMan HCV test (version 2.0, Roche Molecular Systems, Branchburg, NJ, USA). RASs in NS5A and NS3 were investigated using reverse transcription polymerase chain reaction and population sequencing of the corresponding genes to guide the selection of salvage DAAs (Quest Diagnostics, Secaucus, NJ, USA). Patients were evaluated for virologic response at weeks 2 and 4 of therapy, at the end of therapy, and at 4 and 12 weeks after completion of therapy. Patients were monitored for adverse events by physical assessment, complete blood cell count, and comprehensive metabolic panel at every clinic visit. Adverse events were graded by the Common Terminology Criteria for Adverse Events grading scale.^5^ Cirrhosis was diagnosed based on liver biopsy or a combination of clinical findings (e.g. ascites, encephalopathy, or jaundice), serum biomarker levels (measured with the Prometheus Fibrospect II assay [Prometheus Laboratories, San Diego, CA, USA]), and radiologic findings (e.g. hepatic nodularity).^6^

## Results

### *General characteristics*

Of the 164 patients enrolled with initial treatment with DAAs, 16 (10%) patients did not achieve SVR, and 11 of these received salvage therapy with the first course of DAAs with or without ribavirin (Table 1). Of the five patients who did not receive salvage therapy, two died before retreatment, one was lost to follow‐up, and two had hepatocellular carcinoma (HCC) and opted to delay retreatment until after the liver transplant.

**Table 1:** Patient characteristics, direct‐acting antiviral regimens, and outcomes

Cancer type	Sex	Age, years	HCV geno	Cirrhosis	Cancer	Prior IFN	Initial DAA regimen	Duration of initial DAA regimen, weeks	NS3 RAS at failure	NS5A/B RAS at failure	First salvage DAA regimen	Presalvage ANC/ALC (cells/μL)	Duration of first salvage regimen, weeks	Chemo within 6 months of first salvage regimen	AEs grade 3 or 4	Virologic outcome
Solid tumor	M	56	1a	Yes	Colon	Yes	SOF + IFN + RBV	12	No	No	LDV/SOF	1520/1640	24	No	No	SVR12
	M	87	1b	Yes	HCC	No	SOF + SIM	12	No	No	LDV/SOF+ RBV	1740/370	24	No	No	Relapse
	M	58	1a	No	HCC	No	LDV/SOF	12	No	Q30Q/H, Y93H	SOF + SIM+ RBV	5480/1860	24	No	No	SVR12
	M	63	1a	Yes	HCC	Yes	LDV/SOF	24	No	Q30R	SOF/VEL+ RBV	3940/1000	24	Sorafenib	No	Relapse†
	M	66	1a	No	SCC oropharynx	No	LDV/SOF	12	Q80K, D168E	M38A, Q30H	ELB/GZV+ RBV	2650/1130	24	No	No	Relapse‡
	M	62	3	No	SCC tonsil	Yes	SOF+ DCV	12	No	Y93H	SOF/VEL/VOX+ RBV	3070/450	12	Cisplatin	No	SVR12
	M	61	2, 4	No	HCC	No	SOF+ RBV	12	No	No	SOF/VEL	6480/1040	12	No	No	SVR12
	M	57	3	No	Melanoma	Yes	SOF+ RBV	12	No	No	SOF/VEL/VOX	4300/1980	12	No	No	SVR12
Hematol Malig	M	55	1a	No	Multiple Myeloma	Yes	SOF + SIM	12	No	No	SOF/VEL/VOX + RBV	2360/1600	12	Carfilzomib, pabinostat	No	SVR12
	F	56	3a	No	DLBCL	Yes	SOF+ RBV	24	No	No	SOF + DCV+ RBV	2710/830	24	No	No	SVR12
	M	65	1a	No	DLBCL, HCC	No	LDV/SOF	12	No	Q30R	SOF + SIM+ RBV	2330/820	24	No	No	Relapse

† The patient received a second salvage DAA regimen (sofosbuvir/velpatasvir/voxilaprevir plus ribavirin for 12 weeks) and achieved SVR12.

‡ The patient received a second salvage DAA regimen (sofosbuvir plus paritaprevir/ritonavir/ombatsavir/dasabuvir plus ribavirin for 24 weeks) and achieved SVR12.

AEs, adverse events; ALC, absolute lymphocyte count; ANC, absolute neutrophil count; Chemo, chemotherapy; DAA, direct‐acting antiviral; DCV, daclatasvir; DLBCL, diffuse large B‐cell lymphoma; ELB/GZV, elbasvir/grazoprevir; EOT; end of treatment; Geno, genotype; HCC, hepatocellular carcinoma; HCV, hepatitis C virus; Hematol, hematologic; IFN, interferon; Malig, malignancy; RAS, resistance‐associated substitution; RBV, ribavirin; SCC, squamous cell carcinoma; SIM, simeprevir; SOF, sofosbuvir; LDV/SOF, ledipasvir/sofosbuvir; SOF/VEL, sofosbuvir/velpatasvir; SOF/VEL/VOX, sofosbuvir/velpatasvir/voxilaprevir; SVR12, sustained virologic response at 12 weeks after end of treatment.

Of the 11 retreated patients, 10 (91%) were men, 6 (55%) HCV genotype 1a, and 5 (45%) had cirrhosis. Eight patients (73%) had solid tumors, and three patients (37%) had hematologic malignancies. HCC was the most common cancer (Table 1). All patients were started on DAA therapy after having their cancer stable or in remission for at least 3–6 months, except in one patient with progressed multiple myeloma who was treated with DAAs to allow access to potentially life‐saving investigational cancer therapy.

### *Efficacy*

Of 11 patients, 7 (64%) achieved SVR at 12 weeks after completion of the first DAA salvage therapy. The four patients without SVR developed viral relapse. Two of them later achieved SVR after undergoing a second DAA salvage therapy. One of these two patients received sofosbuvir/velpatasvir/voxilaprevir plus ribavirin for 12 weeks, and the other received sofosbuvir plus paritaprevir/ritonavir/ombatsavir/dasabuvir for 24 weeks. The overall SVR rate in our study was 82% (9 of 11 patients). Most of them (seven of nine patients or 78%) had undetectable viral load at week 4 of DAA treatment, and all had undetectable viral at the end of therapy. In the entire cohort, 4 out of 11 patients received sofosbuvir, velpatasvir, and voxilaprevir with or without ribavirin, 3 as a first salvage regimen, and 1 as second salvage treatment and all of them (100%) achieved SVR12.

The four patients without SVR after first salvage therapy, had genotype 1a disease, and HCC, with one patient having concurrent diffuse large B‐cell lymphoma and HCC.

### *Viral resistance testing*

Five patients had RASs detected prior to first DAA salvage therapy, and four of these patients (80%) achieved an SVR (two of the four patients received second salvage therapy). Of the four patients who did not achieve SVR after first salvage, two patients had developed a Q30 NS5A mutation, and one patient had developed Q80 NS3 and Q30 NS5A mutations after initial DAA therapy. Two of the patients without SVR after first salvage (50%) were noted to have an absolute lymphocyte count (ALC) of less than 1000 cells/μL, compared to 29% of those who achieved SVR after first salvage. None of the patients had an absolute neutrophil count (ANC) less than 1500 cells/μL prior to starting salvage therapy.

### *Safety*

Seven patients developed grade 1 or 2 adverse events (fatigue, headache, skin dryness, and shortness of breath in one patient each; anemia in six patients). No patient reported grade 3 or 4 adverse events while receiving salvage therapy. No patient required interruption of DAA therapy because of clinical or laboratory abnormalities. Of the seven patients treated with a ribavirin‐containing regimen, six (86%) experienced grade 1 or 2 anemia. Ribavirin was discontinued prior to the planned end of treatment in two patients with grade 2 anemia. No clinically significant drug–drug interactions were observed.

## Discussion

To our knowledge, this is the first prospective study describing the efficacy and safety of DAA salvage therapy in HCV‐infected patients with cancer without an SVR after DAAs. The low rate of treatment failure to initial DAA therapy, 10% (16/164), is encouraging. The rates of SVR after first and second DAA salvage therapies in this study, 64%, and 82%, respectively, are slightly lower than the SVR rates of 88–96% after DAA salvage therapy reported in HCV‐infected patients without cancer.^3^, ^7^, ^8^ The lower SVR rate observed in our series is likely related to the immunocompromised condition of our patients and the fact that several of them were treated with regimens, which are now not considered the first‐line salvage therapy. However, it is encouraging that 82% of patients were able to be cured virologically.

Cirrhosis, HCC, the presence of RAS, and low ALC were all risk factors for HCV relapse after DAA therapy.^1^, ^9^ We found no clear evidence of more RAS development in our series than in other series of HCV‐infected patients without cancer.^2^ The Q30 NS5A RAS is associated with resistance to all NS5A inhibitors except velpatasvir and was found, in one study, to develop in 71% of patients treated with the combination of daclatasvir and sofosbuvir and 52% of patients treated with the combination of ledipasvir/sofosbuvir.^2^ The Q30 NS5A RAS was seen in every patient with genotype 1a infection in our cohort without SVR after the first salvage therapy. However, the majority of patients in our study with previously existing mutations responded successfully, indicating that the presence of RAS prior to salvage is not a major barrier to achieving SVR.

A study of 26 patients without SVR after initial DAA therapy retreated with sofosbuvir, grazoprevir, elbasvir, and ribavirin showed an SVR rate of 96% despite the presence of baseline NS5A RAS in 92% of patients.^8^ A recent trial of the efficacy of DAA therapy with the combination regimens sofosbuvir/velpatasvir/voxilaprevir in DAA‐experienced patients led to their approval for salvage therapy given the high rates of SVR (96–98%) among patients across HCV genotypes.^7^ In our study, four patients were treated with the combination of sofosbuvir/velpatasvir/voxilaprevir with or without ribavirin, and they all responded (SVR 12100%). Glecaprevir/pibrentasvir has also been shown to achieve SVR rates of 89–96% in patients with HCV genotypes 1–6 infection with viral relapse after prior DAA treatment regardless of the presence of pre‐existing NS5A RAS.^10^, ^11^, ^12^ None of our patients were treated with glecaprevir/pibrentasvir. The regimen of glecaprevir/pibrentasvir plus sofosbuvir and ribavirin has been shown to have an SVR rate of 96% in patients who had previously failed glecaprevir/pibrentasivir alone.^12^ This regimen may be another option for our cancer patients requiring DAA salvage therapy.

Our study has several limitations. The most significant limitation is the small sample size, which precluded statistical analysis of our data. In addition, two patients in our cohort who did not have SVR after initial DAA therapy were retreated with the combination of sofosbuvir, simeprevir, and ribavirin, a combination no longer recommended as a first‐line salvage regimen for DAA‐experienced patients.^1^

In conclusion, DAA salvage therapy in HCV‐infected cancer patients without SVR after initial DAA therapy is associated with a reasonable success rate for an immunocompromised population and has a low incidence of adverse effects. The efficacy seems to be better with newer antivirals, but larger studies with recently approved DAA salvage therapies are needed to eliminate HCV infection in difficult‐to‐treat cancer patients.
